# D-Galacturonic acid reduction by *S. cerevisiae* for L-galactonate production from extracted sugar beet press pulp hydrolysate

**DOI:** 10.1007/s00253-021-11433-5

**Published:** 2021-07-16

**Authors:** J. Wagner, D. Schäfer, N. von den Eichen, C. Haimerl, S. Harth, M. Oreb, J. P. Benz, D. Weuster-Botz

**Affiliations:** 1 Institute of Biochemical Engineering, Technical University of Munich, Boltzmannstr. 15, D-85748 Garching, Germany; 2 Faculty of Biological Sciences, Institute of Molecular Biosciences, Goethe University Frankfurt, Max-von-Laue Straße 9, D-60438 Frankfurt am Main, Germany; 3 TUM School of Life Sciences, Professorship of Fungal Biotechnology in Wood Science, Technical University of Munich, Hans-Carl-von-Carlowitz-Platz 2, D-85354 Freising, Germany

**Keywords:** Extracted sugar beet press pulp, Pectin, Enzymatic hydrolysis, D-Galacturonic acid, L-Galactonate, *Saccharomyces cerevisiae*

## Abstract

**Abstract:**

Pectin-rich residues are considered as promising feedstocks for sustainable production of platform chemicals. Enzymatic hydrolysis of extracted sugar beet press pulp (SBPP) releases the main constituent of pectin, d-galacturonic acid (d-GalA). Using engineered *Saccharomyces cerevisiae*, d-GalA is then reduced to l-galactonate (l-GalOA) with sorbitol as co-substrate. The current work addresses the combination of enzymatic hydrolysis of pectin in SBPP with a consecutive optimized biotransformation of the released d-GalA to l-GalOA in simple batch processes in stirred-tank bioreactors. Process conditions were first identified with synthetic media, where a product concentration of 9.9 g L^-1^ L-GalOA was obtained with a product selectivity of 99% (L-GalOA D-GalA^-1^) at pH 5 with 4% (w/v) sorbitol within 48 h. A very similar batch process performance with a product selectivity of 97% was achieved with potassium citrate buffered SBPP hydrolysate, demonstrating for the first time direct production of L-GalOA from hydrolyzed biomass using engineered *S. cerevisiae*. Combining the hydrolysis process of extracted SBPP and the biotransformation process with engineered *S. cerevisiae* paves the way towards repurposing pectin-rich residues as substrates for value-added chemicals.

**Key points:**

• *Efficient bioreduction of D-GalA with S. cerevisiae in stirred-tank reactors*

• *Batch production of L-GalOA by engineered S. cerevisiae with high selectivity*

• *Direct L-GalOA production from hydrolyzed sugar beet press pulp*

**Graphical abstract:**

**Figure:**
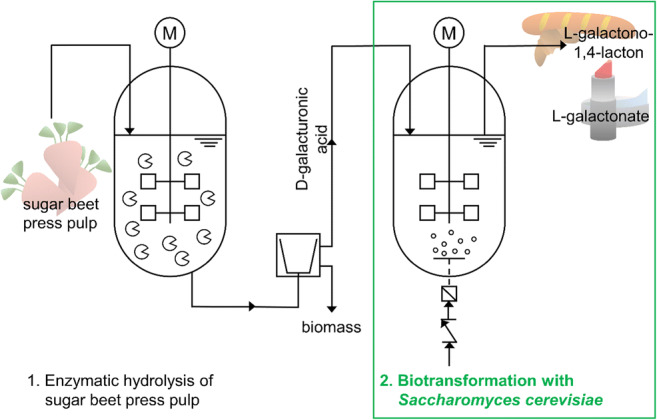
Bioreduction of D-galacturonic acid to L-galactonate with recombinant Saccharomyces cerevisiae enables for the first time the valorization of hydrolysates from extracted sugar beet press pulp for the sustainable production of value-added chemicals.

## Introduction

The bioconversion of agricultural residues into second-generation biofuels has been well studied and extensively reviewed (Kuivanen et al. 2019; Hortsch and Corvo 2020; Jansen et al. 2017; Lin and Tanaka 2006). The hydrolysis of lignocellulosic plant residues is industrially established, and hydrolysates are used for bioethanol production (Cardona and Sánchez 2007; Erdei et al. 2012; Gírio et al. 2010). Plant residues rich in pectin, such as extracted citrus fruits or sugar beet pulps, have sparked interest in recent years as novel feedstocks with high potential for bioconversions (Edwards and Doran-Peterson 2012). More than 250 million tons of citrus fruits and sugar beets are harvested annually for juicing and sugar refining (FAO 2020; Kuivanen et al. 2019). The residual dry pulp consists of 20–40% (w/w) pectin, a heteropolysaccharide comprised mainly of an α-1,4-linked d-galacturonic acid backbone (Mohnen 2008). Depending on the pectic subtype, d-GalA is alternated with l-rhamnose monomers in the backbone, additionally being substituted with different mono- and oligosaccharide side chains (Micard et al. 1996; Schmitz et al. 2019). Usually, the extracted and dried pulps are used energetically or as animal feed additive (Richard and Hilditch 2009; Zema et al. 2018).

To access monomeric d-GalA from pectic residues, either chemical or enzymatic pre-treatment is necessary. Chemical pre-treatment seems favorable for degradation of the strong cellulose and hemicellulose network surrounding the pectin in plant cell walls. However, formation of non-fermentable and toxic byproducts for microorganisms during chemical hydrolysis, like furfural or hydroxymethylfurfural (HMF), favors the utilization of enzymes for the degradation of pectic residues (Palmqvist and Hahn-Hägerdal 2000). Though less efficient compared to the chemical breakdown, fewer potentially inhibitory components are formed (Martins et al. 2020). Natively, fungi can hydrolyze this complex heteropolysaccharide with a toolbox of different pectinase enzymes (Garg et al. 2016; Hassan et al. 2019; Kashyap et al. 2001; Jayani et al. 2005). Leijdekkers et al. (2013) reported a hydrolysis yield of 79% resulting in 11.8 g L^-1^ d-GalA after 48 h from extracted sugar beet press pulp using different commercially available enzyme mixtures. Recently, non-optimized *Aspergillus niger* strains were evaluated for their pectinase activity to depolymerize different pectinaceous agricultural residues (Schäfer et al. 2020). The resulting enzyme cocktail was used for sodium acetate buffered hydrolysis of extracted sugar beet press pulp at pH 4.5 in a batch process. In total, 8.0 g L^-1^ of monomeric d-GalA could be released within 48 h with a yield of 36.4% (w/w).

Recent approaches to valorize low-value pectin-rich residues used microorganisms to produce higher value products from d-GalA. While Kuivanen et al. (2012) exploited deletion mutants of *A. niger* for the reduction of d-GalA in batch processes to produce up to 8.7 g L^-1^ of l-galactonate (l-GalOA) in 144 h, Protzko et al. (2018) used recombinant *S. cerevisiae* for the oxidation of d-GalA. Up to 8 g L^-1^ of *meso-*galactaric acid were formed by the yeast cells in batch processes with glucose as co-substrate. Both biotransformation products have potential applications as a pharmaceutical conjugate or chelator in the industry (Kuivanen et al. 2019). Especially l-GalOA, being physico-chemically similar to d-gluconic acid, may be applied as an alternative chelating agent in the cosmetic and food industries (Kuivanen et al. 2012). Its lactonic form, l-galactono-1,4-lactone, is the precursor of vitamin C (Kuivanen et al. 2015; Sauer et al. 2004) and an analog of glucono-δ-lactone (E575), an acidifier applied in various foodstuffs. While the application of filamentous fungi natively producing l-GalOA might appear advantageous, the yeast *S. cerevisiae*, being the microbial workhorse of bio-industry (Hong and Nielsen 2012; Nielsen et al. 2013), surpasses the fungus in fast genetic manipulation for metabolic engineering. Furthermore, high growth rates resulting in shorter fermentation processes and the production of utilizable byproducts such as ethanol lower operating costs and the risk of potential contamination by other microbes (van Maris et al. 2006).

*S. cerevisiae* has been engineered for the reduction of d-GalA, given that this yeast is natively unable to metabolize d-GalA (van Maris et al. 2006). One auspicious metabolic engineering approach was the expression of transporters from *A. niger* or *Neurospora crassa* in *S. cerevisiae* enabling the efficient uptake of d-GalA in yeast cells (Benz et al. 2014; Harth et al. 2020; Protzko et al. 2018). Consecutively, d-GalA is reduced with overexpressed NAD(P)H-dependent oxido-reductases in the yeast cells (Richard and Hilditch 2009). Biz et al. (2016) demonstrated the consumption of only 1 g L^-1^ d-GalA with engineered yeast strains expressing four genes of the reductive d-GalA pathway from *A. niger* to produce ethanol in the presence of 80 g L^-1^ fructose in 24 h. The low conversion of d-GalA was ascribed to the higher oxidation state of d-GalA compared to other hexoses, such as fructose, and an intracellular lack of reducing factors. Conclusively, the authors suggested the introduction of enzymes to regenerate NAD(P)H in a stoichiometric manner for d-GalA reduction. This hypothesis is further supported by Protzko et al. (2018), who showed preferred expression of the NAD(P)H utilizing d-GalA reductase GaaA from *A. niger* in *S. cerevisiae* in enrichment experiments with d-GalA as the sole carbon source. Jeong et al. (2020) approached this assumed lack of reducing equivalents by expressing enzymes from *Ambrosiozyma monospora*, *Pichia stipitis*, and *T. reesei* for the co-utilization of the pentoses xylose and arabinose in yeast strains carrying different fungal enzymes from *A. niger* and *T. reesei* for the reduction of d-GalA. Fermentation of these sugars involves NAD(P)H generating pathways, and growth of engineered *S. cerevisiae* was made possible with consumption of 33.7 g L^-1^ xylose, 25.9 g L^-1^ arabinose, and 15.3 g L^-1^ d-GalA as the sole carbon sources, thereby overcoming the redox problem of d-GalA catabolism in yeast. Lastly, Harth et al. (2020) described a fully functional, redox-balanced enzyme system in *S. cerevisiae* expressing the d-galacturonic acid transporter from *A. niger*, AnGar1, an NADPH-dependent reductase from *A. niger*, for the reduction of d-GalA to l-GalOA and an NADP-dependent sorbitol dehydrogenase from *Yarrowia lypolytica* (YlSdr) with sorbitol as the co-substrate to regenerate the reducing factors. In this constellation, NADPH is regenerated when sorbitol is oxidized to fructose achieving a nearly complete conversion of d-GalA into 4.4 g L^-1^ l-GalOA within 8 days in batch processes with synthetic medium (Harth et al. 2020).

With the engineered *S. cerevisiae* strain of Harth et al. (2020) at hand (*S. cerevisiae* SiHY001), the utilization of enzymatically hydrolyzed pectinaceous residues for l-GalOA production seems feasible. Still, process conditions that favor the enzymatic hydrolysis of sugar beet press pulp (SBPP) with pectinases from *A. niger*, as described by Schäfer et al. (2020), do not necessarily support biotransformation conditions for the recombinant yeast strain. Especially, the unique composition and processing of pectinaceous hydrolysates may bear unknown inhibitory substances for yeast (Martins et al. 2020). Therefore, we firstly studied the effects of pH, initial sorbitol, and d-GalA concentrations on l-GalOA production with *S. cerevisiae* SiHY001 in simple batch processes in controlled stirred-tank bioreactors with defined medium to adapt the bioreduction process to the previous enzymatic hydrolysis (Schäfer et al. 2020). Secondly, we adapted the enzymatic hydrolysis process of SBPP for d-GalA release to enable a direct subsequent whole-cell biotransformation with *S. cerevisiae* SiHY001.

## Materials and methods

Unless stated otherwise, all chemicals were purchased from Carl Roth GmbH or Sigma-Aldrich.

### Microbial strains

Biotransformation of d-GalA to l-GalOA was studied with *S. cerevisiae* SiHY001, which was earlier described to be able to reduce d-GalA to l-GalOA with sorbitol as co-substrate for growth and cofactor regeneration (Harth et al. 2020). *S. cerevisiae* SiHY001 contains expression cassettes with the genes for (i) the endogenous sorbitol transporter HXT13, (ii) the sorbitol dehydrogenase YlSdr (UniProtKB—Q6CEE9) from *Yarrowia lipolytica*, (iii) the d-galacturonic acid reductase AnGar1 (UniProtKB-A2R7U3) from *A. niger*, as well as (iv) the d-galacturonic acid transporter AnGatA (UniProtKB—A2R3H2) from *A. niger*. Cassettes were integrated into the *URA3* locus of the parental strain *S. cerevisiae* EBY.VW4000, an auxotrophic, hexose-transporter deficient strain (Wieczorke et al. 1999). The cells were maintained in 25% (v/v) glycerol stocks at – 80 °C with a cell dry weight (CDW) concentration of 0.5 g L^-1^ (Table 1).

**Table 1:** Genotype of the parental *S. cerevisiae* EBY.VW4000 and the engineered *S. cerevisae* SiHY001 used in this study. Prefixes “p” and “t” indicate promoters and terminators for genes, respectively

*S. cerevisiae*	Genotype	Reference
EBY.VW4000	*MATa leu2-3,112 ura3-52 trp1-289 his3-1 MAL2-8c SUC2 Δhxt1-17 Δgal2 Δstl1::loxP Δagt1::loxP Δmph2::loxP Δmph3::loxP*	(Wieczorke et al. 1999)
SiHY001	**EBY.VW4000** *Δura3::pCCW12-AnGATA-tPGK1-pPGK1-AnGAR1-tENO1-pTDH3-HXT13-tSSA1-pTEF2-YlSDR-tADH1-pAgTEF-kanMX-tAgTEF*	(Harth et al. 2020).

### Seed cultures

For the inoculation of stirred-tank bioreactors, seed cultures were grown 72 h in autoclaved (121 °C, 20 min) 1000-mL shake flasks with 100 mL of SC medium containing 1.7 g L^-1^ BD™ Difco™ yeast nitrogen base without amino acids and ammonium sulfate (Fisher Scientific GmbH, Schwerte, Germany) and sterile filtered (0.22 μm, Steritop, Merck KGaA, Darmstadt, Germany) amino acid mix (0.056 g L^-1^ adenine, 0.192 g L^-1^ arginine, 0.192 g L^-1^ methionine, 0.072 g L^-1^ tyrosine, 0.288 g L^-1^ isoleucine, 0.323 g L^-1^ lysine*H_2_O, 0.240 g L^-1^ phenylalanine, 0.288 g L^-1^ valine, 0.288 g L^-1^ threonine, 0.096 g L^-1^ uracil, 0.096 g L^-1^ histidine, 0.095 g L^-1^ tryptophan, 0.288 g L^-1^ leucine), 10 g L^-1^ ammonium sulfate, 10 g L^-1^ sorbitol, and 5 g L^-1^ D-galacturonic acid at 30 °C and 180 rpm (Multitron, Infors HT, Bottmingen, Switzerland). Shake flasks were inoculated with 500 μL glycerol stocks of *S. cerevisiae* strain SiHY001.

### Preparation of inoculum for stirred-tank bioreactors

Inoculums for stirred-tank bioreactors were prepared by centrifuging cells from seed cultures at 3000 g within 10 min at 4 °C (Rotixa 50 RS, Andreas Hettich GmbH & Co.KG, Tuttlingen, Germany) and washing the pellet twice with sterile phosphate buffered saline (8 g L^-1^ NaCl, 0.2 g L^-1^ KCl, 1.44 g L^-1^ Na_2_HPO_4_, 0.24 g L^-1^ KH_2_PO_4_, pH 7.4). After re-suspension in 5 mL sterile SC medium, bioreactors were inoculated with an amount equivalent to 0.25 g L^-1^ cell dry weight (CDW).

### Medium preparation

The medium for pH variations was identical to the medium of the seed cultures. For batch processes with varying medium composition, 17 g L^-1^ solid medium component (BD™ Difco™ yeast nitrogen base without amino acids and ammonium sulfate, Fisher Scientific GmbH, Schwerte, Germany) was used with a two-fold of amino acid concentrations as described in 2.2. Buffer sensitivity experiments were performed in identical medium as seed cultures, except that pH adjusted buffers (1 M, pH 5) substituted aqueous proportions of the medium for the respective concentrations of sodium acetate, citrate potassium acetate, or citrate.

Extracted, pre-dried, and milled SBPP with a particle size < 1 mm (Südzucker AG, Obrigheim, Germany) was hydrolyzed with an enzyme mix produced from cultivation with *A. niger.* Methods for pectinase production and pectinase (PGase) activity measurements are described in detail by Schäfer et al. (2020). For hydrolysis of residues, 70 g L^-1^ SBPP suspended in 100 mM potassium citrate buffer was autoclaved (121 °C, 20 min). Sterile-filtered fermentation supernatant containing the produced enzyme mixture equivalent to 57 U PGase/g SBPP was added afterwards together with kanamycin (34 μg mL^-1^). Batch hydrolysis of 5 L SBPP suspension was performed in stirred-tank reactors (Labfors 3, Infors HT, Bottmingen, Switzerland) controlled at pH 4.5, 30 °C and continuous stirring with two 3-blade segment impellers with 65 mm in diameter (Infors HT, Bottmingen, Switzerland) at 700 min^-1^ for 169 h. The hydrolysate was harvested by centrifugation for 60 min at 3260 rcf, and subsequently sterile-filtrated applying filters with different pore sizes of 2.5 μm, 0.45 μm, and 0.22 μm (Whatman™ filter paper grade 5, 55 mm, GE Healthcare Life Science, Freiburg, Germany, and Millipore Express® PLUS 0.45 μm or 0.22 μm PES, 47 mm, Merck Millipore, Darmstadt, Germany).

### Batch processes in parallel stirred-tank bioreactors at 10 mL-scale

Sterile single-use bioreactors with baffles and with DO and pH sensors (BZ1002, 2mag AG, Munich, Germany and sensor spots for DO (PST3-HG) and pH (LG1), PreSens Precision Sensing GmbH, Regensburg, Germany) were operated with gas-inducing stirrers and 10 mL reaction volume in a parallel bioreactor system (bioreactor48, 2mag AG, Munich, Germany), embedded in a liquid handling system for sampling (MICROLAB STAR^TM^LetM, Hamilton Bonaduz AG, Switzerland). Immobilized sensors for DO and pH monitoring were equilibrated at least 30 min with sterile medium prior to sterile inoculation of each bioreactor. Batch processes were performed at 30 °C and 0.1 L min^-1^ of sterile air with an impeller speed of 2000 rpm. Evaporation compensation was achieved by setting the headspace cooling of the reactors to 20 °C and pre-saturating the inlet air with water.

### Batch processes in parallel stirred-tank bioreactors at 600 mL-scale

Batch processes were performed with a parallelized lab-scale stirred-tank bioreactor system (DASGIP® Parallel Bioreactor System, Eppendorf AG, Hamburg, Germany) applying a working volume of 0.6 L. Each stirred-tank reactor was equipped with probes for temperature, dissolved oxygen (DO) concentration, and pH, as well as feed lines for pH control (1 M KOH, 0.5 M H_2_SO_4_), 2 six-blade Rushton turbines, and a gas mixing unit. Waste gas concentrations of O_2_ and CO_2_ were monitored separately online with a gas analyzer (EasyLine, ABB, Zurich, Switzerland) for each reactor. Prior to inoculation, two-point DO calibration was performed at 2 vvm and 600 rpm by stripping the medium first with nitrogen gas until DO = 0% air saturation was achieved and afterwards with air until DO = 100% air saturation was achieved in the fermentation medium at 30 °C. Aeration, temperature, and pH were kept constant at 0.5 vvm, 30 °C and pH 5 except for pH variation experiments. DO concentration was kept above 30% air saturation by the controlled increase of the stirrer speed from initially 200 rpm to 800 rpm at the maximum.

### Offline analytics

Optical density for the determination of cell densities was performed at a wavelength of 600 nm (OD_600_) in 10-mm cuvettes using a single beam photometer (Genesys 10S UV–VIS, Thermo Scientific, Neuss, Germany) or with a microplate reader (Multiscan^TM^ FC, Thermo Fisher Scientific, Waltham, USA). Values obtained from the microplate reader (Multiscan^TM^ FC, Thermo Fisher Scientific, Waltham, USA) were correlated to the single beam photometer (OD_600_) with a second order polynomial (OD_600_ = − 3.6522 x^2^ + 3.975 x + 0.0032; R^2^ = 0.9998).

For the determination of cell dry weight (CDW) concentrations, pre-dried (24 h, 80 °C) and pre-weighted reaction tubes were filled with 2 mL cell suspension and centrifuged for 10 min at 13,000 g. The supernatant was discarded, and the cell pellet dried for 24 h at 80°C. CDW was determined gravimetrically and correlated to optical densities (OD_600_), resulting in a linear correlation factor of 0.61 g L^-1^.

Maximum growth rates were estimated by linear regression after plotting the logarithm of the CDW concentrations during exponential growth as a function of the process time.

Metabolites in the fermentation broth were determined with high-performance liquid chromatography (Agilent 1100, Agilent Technologies Inc., Santa Clara, USA or Shimadzu Prominence-i LC-2030C Plus, Shimadzu Scientific Instruments, Inc., Japan) equipped with an RI detector (Agilent 1200, Agilent Technologies Inc., Santa Clara, USA or Shimadzu RID-20A, Shimadzu Scientific Instruments, Inc., Japan), an UV detector at 210 nm (integrated in Shimadzu Prominence-i LC-2030C Plus, Shimadzu Scientific Instruments, Inc., Japan), and a thermostat. Samples for analysis were centrifuged for 10 min at 13,000 g and filtered (0.22 μm pore size, Chromafil RC20/15 MS, Macherey-Nagel GmbH & Co.KG, Düren, Germany). Separation of substrates was achieved with an Aminex HPX-87H column (Biorad, Munich, Germany) at 60 °C using a flow rate of 0.5 mL min^-1^ mobile phase with 5 mM sulfuric acid.

## Results

### Biotransformations at varying pH

**Fig. 1:**
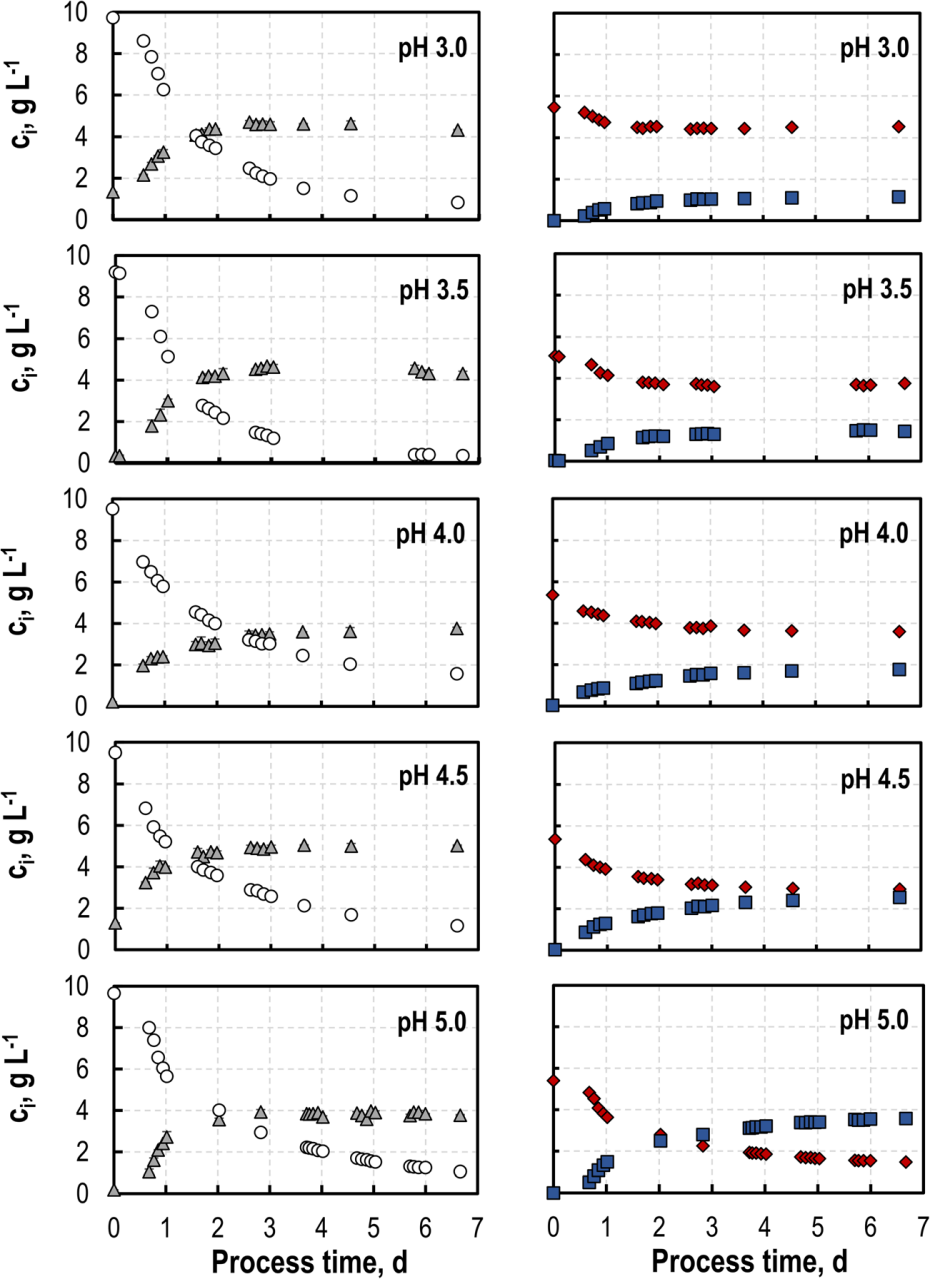
Biotransformation studies with *S. cerevisiae* SiHY001 at varying pH. Concentrations of sorbitol (circle), d-GalA (diamond), l-GalOA (square) and CDW (triangle) in batch operated stirred-tank bioreactors as a function of process time (V = 600 mL, T = 30 °C, DO > 30% air saturation, F_air_ = 0.5 vvm)

Pectinolytic enzymes favor acidic pH for hydrolysis of pectinaceous residues (Kashyap et al. 2001). To avoid a different pH for hydrolysis and biotransformation, five standardized batch processes were performed with *S. cerevisiae* SiHY001 at pH set points between pH 3 and pH 5 with SC medium in stirred-tank bioreactors at 600 mL-scale. Initial d-GalA concentrations of 5 g L^-1^ were chosen based on the reported d-GalA concentrations measured after enzymatic hydrolysis (Schäfer et al. 2020). While similar final biomass concentrations of 4.1–5.0 g L^-1^ CDW were observed at all pH set points, final l-GalOA concentrations varied greatly between 1.1 and 3.6 g L^-1^ (Fig. 1). The highest product concentration and product yield Y_P/Sorbitol_ was observed at pH 5 with 3.6 g L^-1^ l-GalOA, and 0.49 ± 0.04 mol l-GalOA mol^-1^ sorbitol, respectively. A nearly stoichiometric biotransformation yield Y_P/E_ was observed within the estimation error (0.92–1.02 mol l-GalOA mol^-1^ d-GalA) independent of the pH (Table 2), indicating the absence of any further metabolism of either d-GalA or l-GalOA by the yeast cells.

**Table 2:** Biomass concentrations c_X_, l-GalOA concentrations c_P_, l-GalOA yields Y_P/Sorbitol_, and l-GalOA biotransformation yields Y_P/E_ in batch processes with *S. cerevisiae* SiHY001 at varying pH set-points after 160 h (stirred-tank reactors, V = 600 mL, T = 30 °C, DO > 30% air saturation, c_Sorbitol,0_ = 10 g L^-1^, c_d-GalA,0_ = 5 g L^-1^; F_air_ = 0.5 vvm). Different letters indicate significant differences within the displayed data groups (p < 0.05) using a one-way ANOVA followed by a Tukey’s post hoc test

pH	c_X_ g L^-1^	c_P_ g L^-1^	Y_P/Sorbitol_, mol mol^-1^	Y_P/E_, mol mol^-1^
**3.0 (a)**	4.07 ± 0.35	1.32 ± 0.20^de^	0.14 ± 0.00^de^	1.01 ± 0.08
**3.5 (b)**	4.53 ± 0.30	1.46 ± 0.02^de^	0.19 ± 0.02^de^	1.02 ± 0.07
**4.0 (c)**	4.66 ± 1.26	1.98 ± 0.29^e^	0.23 ± 0.00^de^	0.93 ± 0.08
**4.5 (d)**	5.04 ± 0.03	2.70 ± 0.21^abe^	0.35 ± 0.04^abce^	1.00 ± 0.08
**5.0 (e)**	4.56 ± 0.96	3.75 ± 0.33^abcd^	0.49 ± 0.04^abcd^	0.92 ± 0.01

At all pH set points studied, growth of *S. cerevisiae* SiHY001 was finished after ~ 3 days and the remaining sorbitol was further consumed by the resting cells (maintenance metabolism). No biotransformation activities were observed with the resting cells at pH < 4.0, whereas considerable biotransformation activity was observed at pH 4.5 and pH 5.0 (Fig. 1) in the stationary phase. Notably, the growth-coupled biotransformation activity of *S. cerevisiae* SiHY001 contributed predominantly to l-GalOA production (85% at pH 4.5, and 79% at pH 5.0, respectively). Based on the observed best biotransformation results with *S. cerevisiae* SiHY001 at pH 5.0, this pH was chosen as the set point for further studies.

### Biotransformations with varying initial sorbitol concentrations

**Fig. 2:**
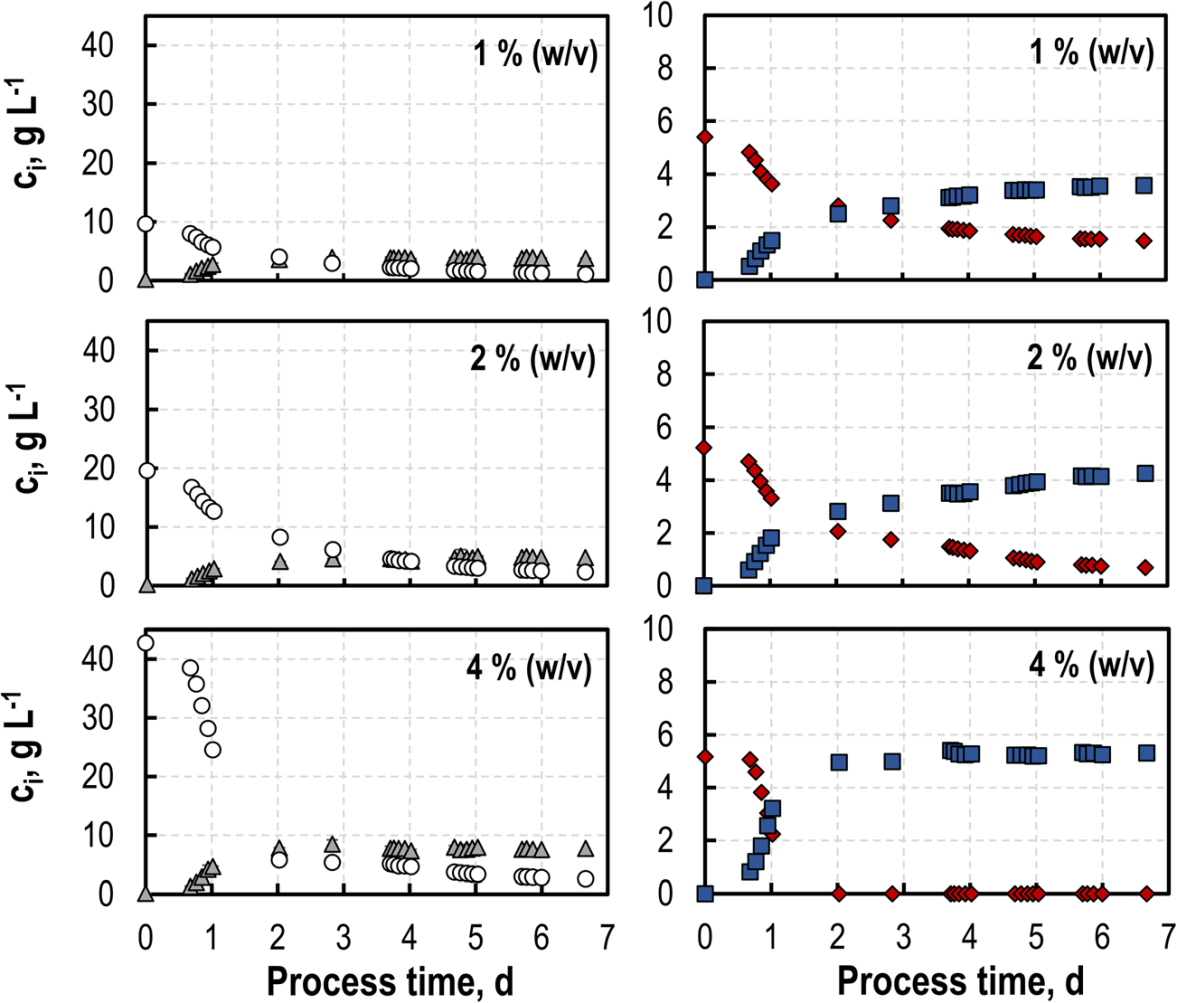
Biotransformation studies with *S. cerevisiae* SiHY001 at varying initial sorbitol concentrations. Concentrations of sorbitol (circle), d-GalA (diamond), l-GalOA (square) and CDW (triangle) in batch operated stirred-tank bioreactors as function of process time (V = 600 mL, T = 30 °C, DO > 30% air saturation, F_air_ = 0.5 vvm)

To avoid any co-substrate limitations in the batch biotransformation processes, three standardized batch processes were performed with *S. cerevisiae* SiHY001 with increasing initial sorbitol concentrations at pH 5 with SC medium in stirred-tank bioreactors at 600 mL-scale keeping the initial d-GalA concentration constant at 5.2 g L^-1^. At 1% (w/v) sorbitol, a final biomass concentration of 3.8 g L^-1^ was reached compared to 4.8 g L^-1^ and 7.6 g L^-1^ at 2% (w/v) and 4% (w/v) sorbitol, respectively (Fig. 2). The final product concentrations were improved as well with increasing initial sorbitol concentrations (3.6 g L^-1^ l-GalOA with 1% (w/v) sorbitol, 4.3 g L^-1^ l-GalOA with 2% (w/v) sorbitol, and 5.2 g L^-1^ l-GalOA with 4% (w/v) sorbitol, respectively). Unfortunately, d-GalA was already consumed after 2 days with 4% (w/v) sorbitol, so that the increased concentration of the *S. cerevisiae* SiHY001 biocatalysts could not be used to full capacity during further batch processing. On the other hand, l-GalOA space-time yield (0.1 g L^-1^ h^-1^) was very much improved with 4% (w/v) sorbitol at full conversion of d-GalA after a process time of 2 days compared to the other batch processes, in which full conversion was not even reached after 7 days (Fig. 2).

Due to the increased consumption of sorbitol for biomass formation with increasing initial sorbitol concentrations, the final l-GalOA yield Y_P/Sorbitol_ decreased from 0.45 mol l-GalOA mol^-1^ sorbitol at the lowest initial sorbitol concentration, to 0.26 mol l-GalOA mol^-1^ sorbitol at twice as much initial sorbitol and to 0.13 mol l-GalOA mol^-1^ sorbitol at the four-fold of initial sorbitol. Additionally, an increased production of non-respiratory metabolites like acetate, glycerol, and especially ethanol was observed with increasing initial sorbitol concentrations (0.0 g L^-1^ EtOH with 1% (w/v) sorbitol, 1.0 g L^-1^ EtOH with 2% (w/v) sorbitol, and 4.1 g L^-1^ EtOH with 4% (w/v) sorbitol, respectively). To avoid any limitation caused by co-substrate supply, further batch processes were performed with 4% initial sorbitol concentration (Table 3).

**Table 3:** Biomass concentrations c_X_, l-GalOA concentrations c_P_, l-GalOA yields Y_P/Sorbitol_, and l-GalOA biotransformation yields Y_P/E_ in batch processes with *S. cerevisiae* SiHY001 at varying initial sorbitol concentrations after 160 h (stirred-tank reactors, V = 600 mL, T = 30 °C, DO > 30% air saturation, pH = 5, c_d-GalA,0_ = 5 g L^-1^; F_air_ = 0.5 vvm)

C_Sorbitol_ g L^-1^	c_X_ g L^-1^	c_P_ g L^-1^	Y_P/Sorbitol_, mol mol^-1^	Y_P/E_, mol mol^-1^
**10**	3.8	3.6	0.45	0.91
**20**	4.8	4.3	0.26	0.94
**40**	7.6	5.2	0.13	1.03

### Growth of *S. cerevisiae* SiHY001 with varying buffer components

**Fig. 3:**
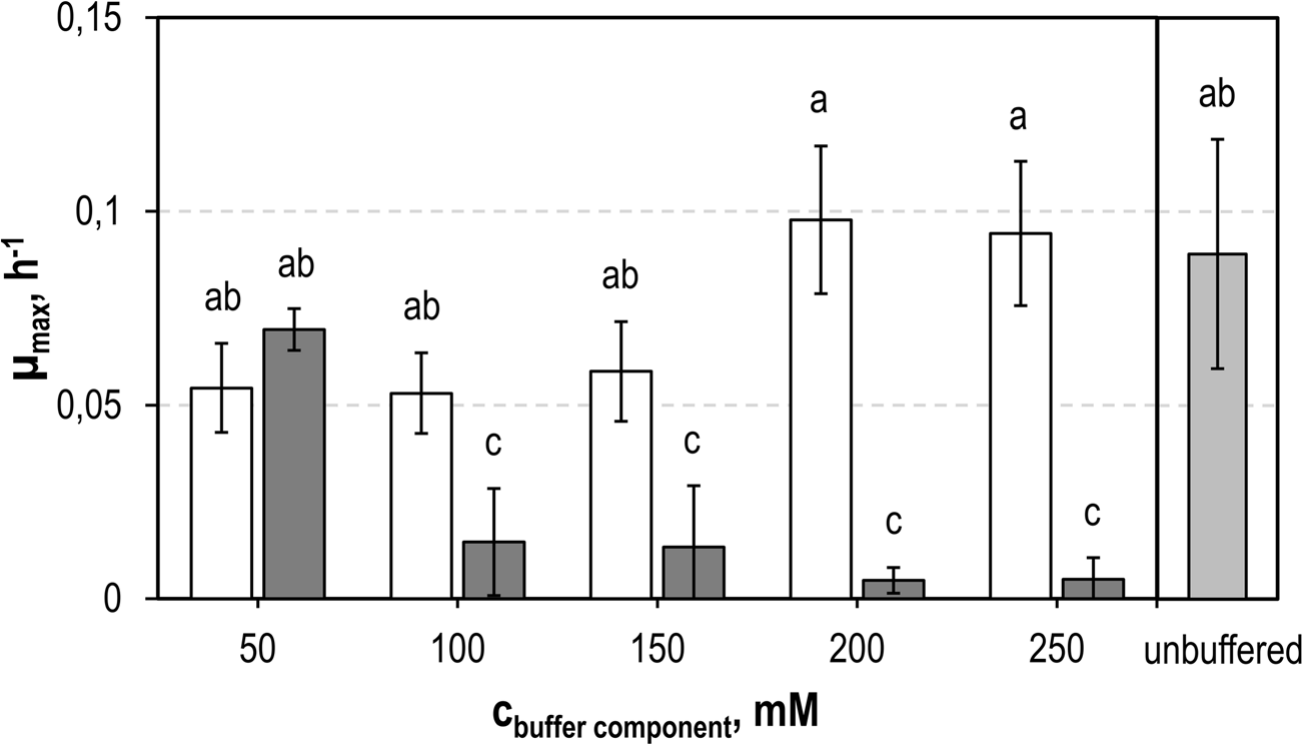
Growth studies with *S. cerevisiae* SiHY001 at varying buffer concentrations. Maximum growth rates in batch operated stirred-tank bioreactors as function of process time with SC medium and 5 g L^-1^ d-GalA and 10 g L^-1^ sorbitol with sodium citrate (gray), potassium citrate (white) and a control (light gray) without buffer(V = 10 mL, pH = 5.0, T = 30 °C, DO > 30% air saturation). *Error bars* represent the standard deviation of three parallel batch experiments. Different letters indicate significant differences within the displayed data groups (p < 0.05) using a one-way ANOVA followed by a Tukey’s post hoc test

The first biotransformation of hydrolyzed SBPP was studied at pH 5 with 4% (w/v) sorbitol in a stirred-tank bioreactor. No growth, no sorbitol consumption, no D-GalA consumption, and no L-GalOA production were observed with *S. cerevisiae* SiHY001 (data not shown). Enzymatic hydrolysis of SBPP had been performed in 100 mM sodium acetate buffer (Schäfer et al. 2020). As acetic acid is known to affect the metabolism of *S. cerevisiae* already at concentrations as low as 3 mM (Sousa et al. 2012), growth of *S. cerevisiae* SiHY001 was studied in miniaturized single-use stirred tank reactors with diverse buffers and buffer concentrations at pH 5.0 in SC medium (sodium acetate, sodium citrate, potassium acetate, and potassium citrate). No growth was observed with acetate buffered media at all concentrations (data not shown). Increasing sodium citrate concentrations resulted in reduced maximum growth rates in batch processes, whereas potassium citrate had no effect on the maximum growth rate at 200 mM and above (Fig. 3). Consequently, the sodium acetate buffer was replaced by 100 mM potassium citrate buffer at pH 5.0 for all further enzymatic hydrolyses of SBPP.

### Biotransformation with sugar beet press pulp hydrolysate

**Fig. 4:**
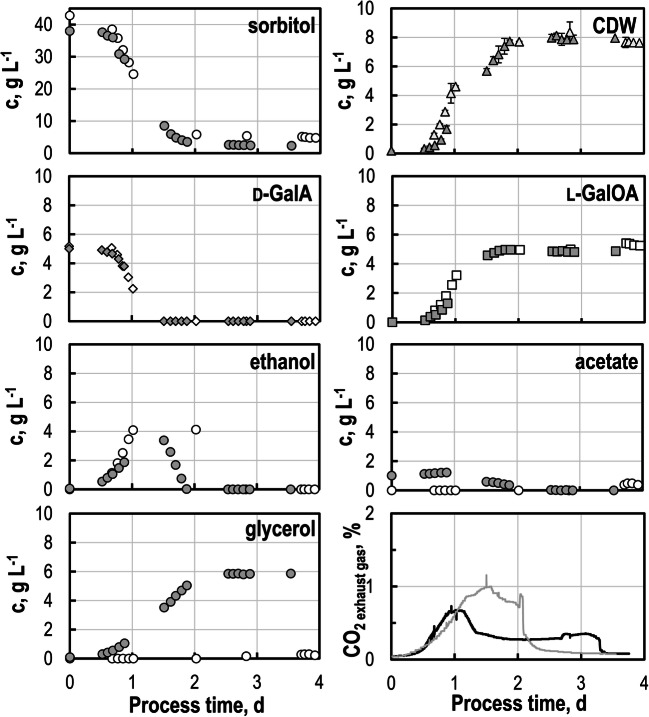
Biotransformation studies with *S. cerevisiae* SiHY001 with sugar beet press pulp (SBPP) hydrolysate (gray) compared to SC medium (white symbols/black line). Concentrations of sorbitol, CDW, d-GalA , l-GalOA, ethanol, glycerol, acetate, and CO_2_ in off-gas as function of the process time in batch operated stirred-tank bioreactors (V = 600 mL, T = 30 °C, DO > 30% air saturation, F_air_ = 0.5 vvm). CDW concentrations were estimated via OD_600_ measurements. *Error bars* represent the min – max values of two individual batch experiments with SBPP hydrolysate in stirred-tank bioreactors and may appear smaller than symbols

SBPP prepared with 100 mM potassium citrate buffer was used for biotransformations at pH 5 in stirred-tank bioreactors. Initial sugar concentrations after medium preparation with SBPP hydrolysate were 40 g L^-1^ sorbitol, 5 g L^-1^ d-GalA, 9.4 g L^-1^ glucose, 13.9 g L^-1^ arabinose, and 3.2 g L^-1^ xylose/mannose/galactose, respectively. Batch process performance of *S. cerevisiae* SiHY001 was very similar to processes with SC medium (Fig. 4). CDW concentrations of 7.9 g L^-1^ were achieved within 2 days. No significant growth could be observed afterwards, although 5.5 g L^-1^ sorbitol were present in SC medium, and 3.0 g L^-1^ sorbitol in SBPP hydrolysate medium, respectively. Biotransformation of d-GalA was already finished within 1.5 days with nearly stoichiometric biotransformation yield Y_P/E_ of 0.99 mol l-GalOA mol^-1^ d-GalA with SC medium and 0.94 ± 0.04 mol l-GalOA mol^-1^ d-GalA with SBPP hydrolysate medium. Product yield Y_P/sorbitol_ was improved with SBPP hydrolysate medium compared to SC medium (0.16 ± 0.01 mol l-GalOA mol^-1^ sorbitol, and 0.13 mol l-GalOA mol^-1^ sorbitol, respectively).

CO_2_ concentrations in the off-gas differed considerably between the biotransformation processes (Fig. 4). SBPP hydrolysate resulted in increased CO_2_ production between a process time of 1–2.2 days. This might result from the consumption of initial acetate (1.0 g L^-1^) provided with SBPP hydrolysate and the re-consumption of ethanol during biotransformation within the first 2 days. Re-consumption of ethanol was very much delayed in the biotransformation with SC medium.

Glycerol accumulation of up to nearly 6 g L^-1^ was only observed with SBPP hydrolysate (Fig. 4), presumably a reaction of *S. cerevisiae* to components of the hydrolysate (Nielsen and Arneborg 2007; Scanes et al. 1998). No fermentation of other hexoses in SBPP hydrolysate, namely galactose and mannose, could be observed due to the strain’s inability to consume these sugars (Wieczorke et al. 1999).

### Biotransformations with increased initial d-GalA concentrations

Theoretically, higher initial d-GalA concentrations of up to 15 g L^-1^ should be accessible after enzymatic hydrolysis of SBPP. As d-GalA reduction by *S. cerevisiae* SiHY001 occurs predominantly growth-coupled and the maximum CDW concentration was around 8 g L^-1^ in batch processes with initial 40 g L^-1^ sorbitol and final sorbitol conversions of 87%, the initial concentrations of the trace elements, vitamins, and salts were increased in biotransformations with 15 g L^-1^ d-GalA to avoid any limitation. Increased initial amino acid concentrations in the medium resulted in no significant alterations of the batch processes with the auxotrophic yeast strain (data not shown).

**Fig. 5:**
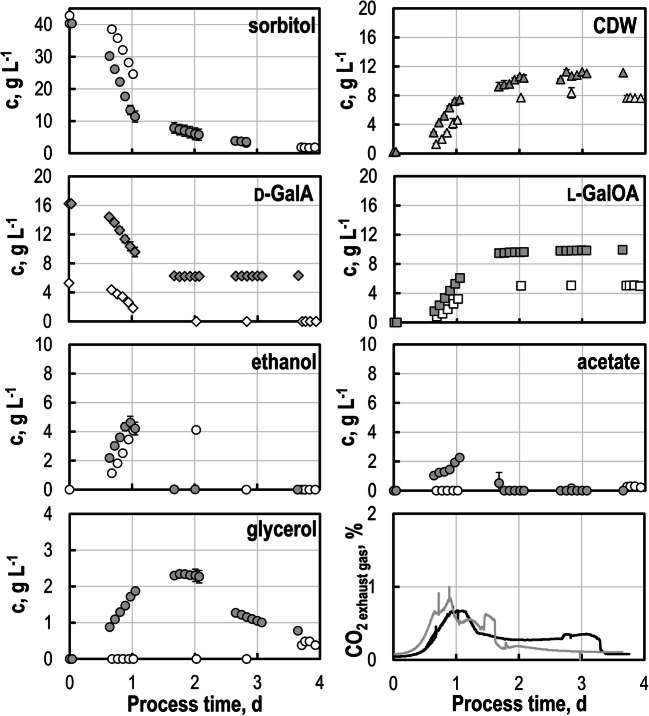
Biotransformation studies with *S. cerevisiae* SiHY001 with increased initial d-GalA concentrations applying 10-fold SC medium (gray) compared to the standard SC medium (white symbols/black line). Concentrations of sorbitol, CDW, d-GalA, l-GalOA, ethanol, glycerol, acetate and CO_2_ in off-gas as function of the process time in batch operated stirred-tank bioreactors (V = 600 mL, T = 30 °C, DO > 30% air saturation, F_air_ = 0.5 vvm). CDW concentrations were estimated via OD_600_ measurements. *Error bars* represent the min – max values of two individual batch experiments with 10-fold SC medium in stirred-tank bioreactors and may appear smaller than symbols

Considerably higher biomass concentrations (11.6 g L^-1^) were achieved with a ten-fold initial increase of the concentrations of trace elements, vitamins, and salts in the SC medium (10-fold SC medium) (Fig. 5). This resulted in an increased product yield Y_P/sorbitol_ (0.28 ± 0.01 mol l-GalOA mol^-1^ sorbitol compared to 0.13 mol l-GalOA mol^-1^ sorbitol) and biomass yield Y_X/sorbitol_ (0.29 ± 0.1 g biomass g^-1^ sorbitol compared to 0.17 g biomass g^-1^ sorbitol). Ethanol and acetate accumulation was increased as well during the first 25 h, but both were readily reconsumed within 24 h thereafter. Glycerol accumulation was solely observed with the 10-fold SC medium up to 2.34 ± 0.03 g L^-1^ and reconsumption of glycerol was not finished after 3.5 days.

Growth-associated biotransformation of d-GalA was improved with 10-fold SC medium. Up to 9.9 ± 0.10 g L^-1^ l-GalOA were formed from 15 g L^-1^ d-GalA (conversion of 61%), but biotransformation stopped after 48 h for unknown reasons. The remaining 7.8 g L^-1^ sorbitol were consumed subsequently for growth of the yeast cells until the batch process was finished after 4 days. This suggests that the initial d-GalA concentrations should not be higher than 10 g L^-1^ to achieve full conversion to l-GalOA within 48 h in the batch biotransformation process with *S. cerevisiae* SiHY001 (Table 4).

**Table 4:** Biomass concentrations c_X_, l-GalOA concentrations c_P_, l-GalOA yields Y_P/Sorbitol_, and biomass yields Y_X/Sorbitol_ in batch processes with *S. cerevisiae* SiHY001 with increased initial D-GalA concentrations and medium components (SC) after 88 h (stirred-tank reactors, V = 600 mL, T = 30 °C, DO > 30% air saturation, pH = 5, c_Sorbitol,0_ = 40 g L^-1^; F_air_ = 0.5 vvm)

Medium	c_X_ g L^-1^	c_P_ g L^-1^	Y_P/Sorbitol_, mol mol^-1^	Y_X/Sorbitol_, g g^-1^
**1-fold SC**	7.7	5.0	0.13	0.17
**10-fold SC**	11.6 ± 0.6	9.9 ± 0.10	0.28 ± 0.1	0.29 ± 0.1

## Discussion

Valorization of pectin-rich residues has moved into focus in recent years. The simultaneous hydrolysis of pectin in extracted SBPP and subsequent direct biotransformation of the released monomer d-GalA would reduce production costs. Enzymatic hydrolysis of pectin in SBPP was reported to be best at pH 4.5 (Schäfer et al. 2020). Although *S. cerevisiae* is known for high tolerance towards low pH (Narendranath and Power 2005) and *S. cerevisiae* growth kinetics are not affected by pH 3.5–6.0 (Carmelo et al. 1996), the best overall performance of the biotransformation of d-GalA to l-GalOA with *S. cerevisiae* SiHY001 was observed at pH 5, the maximum pH under study. The previously suggested passive d-GalA uptake by *S. cerevisiae* at low pH was not observed in our experiments (Souffriau et al. 2012) and low pH did not contribute to enhanced l-GalOA production. d-GalA conversion to ethanol or *meso*-galactaric acid was so far studied solely without pH control in shake flasks (Biz et al. 2016; Jeong et al. 2020; Protzko et al. 2018). For the direct combination of hydrolysis and biotransformation, a compromise is needed between the best pH for enzymatic hydrolysis of the pectin in SBPP (pH 4.5) and the best pH for biotransformation of d-GalA to l-GalOA with *S. cerevisiae* SiHY001 (pH 5.0).

High initial substrate concentrations are necessary in batch biotransformation processes at full conversion to reduce the total costs of biotransformation processes, including downstream processing. At high glucose concentrations, repression of respiration and formation of fermentative by-products is observed with *S. cerevisiae* due to the so-called Crabtree effect (De Deken 1966; Pfeiffer and Morley 2014). Although not specifically described for sorbitol, it was suggested that glycolysis-derived hexose phosphates induce this effect (Lemus et al. 2018). Since sorbitol is oxidized to fructose, and, after phosphorylation, enters glycolysis as fructose-6-phosphate, this might account for the increased non-respiratory behavior of the strain under aerobic conditions. Even with residual 5.8 g L^-1^ sorbitol left in the medium after 48 h (Fig. 2 and Fig. 4), ethanol is consumed. The reasons may be the moderate affinity of the Hxt13 transporter for sorbitol (Jordan et al. 2016) or a self-regulative mechanism of *S. cerevisiae* to avoid inhibiting concentrations of metabolites (Sousa et al. 2012). To the best of our knowledge, batch production of 9.9 ± 0.1 g L^-1^ lGalOA within 48 h (space-time yield of 0.19±0.0 g L^-1^ h^-1^) with 4% (v/w) sorbitol is the highest concentration and space-time yield reported with *S. cerevisiae* in the literature and even exceeds the l-GalOA production with filamentous fungi reported so far (Kuivanen et al. 2014).

*S. cerevisiae* is described to withstand harsh conditions and stresses at the industrial scale (Hong and Nielsen 2012) and has been used for the fermentation of hydrolyzed pectinaceous residues to produce ethanol (Martins et al. 2020). One of the main challenges in the utilization of agro-industrial residues remain the release of weak organic acids and other compounds during hydrolysis (van Maris et al. 2006) that may inhibit growth of *S. cerevisiae*. Enzymatic hydrolysis of pretreated agro-industrial residues, however, requires an adequate buffer system for optimal performance of hydrolytic enzymes. The prior hydrolysis of SBPP with pectinases was initially performed in 100 mM sodium acetate buffer (Schäfer et al. 2020). It has been widely described that the main inhibition of *S. cerevisiae* by acetic acid is its intracellular accumulation (Mira et al. 2010; Sousa et al. 2012). Acetic acid diffuses across the cytoplasm membrane and dissociates into acetate and H_3_O^+^ due to the higher cytosolic pH compared to the extracellular environment, resulting in intracellular acetate accumulation (Pampulha and Loureiro-Dias 1989) and affecting various intracellular mechanisms that lead to reduced or stalled cell growth (Sousa et al. 2012). In contrast, citric acid/citrate does not have this effect on *S. cerevisiae* while it can be growth-inhibiting at higher pH due to its ability to chelate cations (Nielsen and Arneborg 2007). Accordingly, we observed no significant difference in growth rates up to 200 mM potassium citrate buffer in our medium (Fig. 3). Concerning the buffer’s ionic component, sodium affects cell growth negatively when present in the medium. Unlike potassium ions, high extracellular concentrations of sodium ions may disrupt osmotic regulation of the cell and hence induce stress, which results in reduced growth (Ariño et al. 2010; Hohmann 2002).

To the best of our knowledge, SBPP hydrolysate has not been used before for l-GalOA production by recombinant *S. cerevisiae*. Reports on the fermentation of pectin-rich residues such as citrus peel or SBPP were mainly motivated for bioethanol production (Edwards et al. 2011; Martins et al. 2020) or towards whole pectin utilization as carbon source for growth (Huisjes et al. 2012; Jeong et al. 2020; Protzko et al. 2018; Yang et al. 2020). Process performance of *S. cerevisiae* SiHY001 with SBPP hydrolysate was very similar to the performance with synthetic complete medium (SC medium). However, increased production of glycerol could be observed with SBPP hydrolysate and with 10-fold SC medium compared to SC medium. With otherwise identical process conditions, the higher glycerol concentrations cannot be accounted for solely to glycerol being formed as an NADH sink during fermentative growth as a result of the Crabtree effect (Nevoigt and Stahl 1997). More likely, glycerol acts as a response to hyperosmotic stress in 10-fold SC medium (Nevoigt and Stahl 1997). The salty components (sodium chloride, calcium chloride, magnesium sulfate, and potassium phosphate) accumulated in 10-fold SC medium to a total of 0.142 M. Although not as high as reported to induce “mild osmotic stress” for yeast with 0.4 M sodium chloride (Babazadeh et al. 2017), glycerol formation might nevertheless be induced. The much higher glycerol production with SBPP can, however, be explained by an upregulation of proteins involved in glycerol biosynthesis as a stress response of *S. cerevisiae* to citric acid (Lawrence et al. 2004; Nielsen and Arneborg 2007). Further environmental factors might as well synergistically contribute to an overall higher glycerol level with SBPP (Remize et al. 2000). Unlike with synthetic complete medium, 26.5 g L^-1^ non-fermentable monomeric sugars (0.07 M) from SBPP hydrolysate remain in the medium throughout the batch process, since the yeast strain lacks multiple hexose transporter genes to transport these sugars (Wieczorke et al. 1999). Also, a considerable amount of crude protein is present in SBPP hydrolysate (Edwards and Doran-Peterson 2012), potentially acting as nitrogen source that induces glycerol formation (Scanes et al. 2017).

All previous findings show highest product formation during exponential growth and prolonging this phase is therefore desirable*.* Since we were using an auxotrophic strain, the initial approach was to increase the concentration of required amino acids to overcome limitations (Pronk 2002). This did not result in improved biomass formation or biotransformation activity. However, increasing the concentration of vitamins, trace elements, and salts in the medium resulted in both a significantly higher biomass concentration (Fig. 5) and slightly increased growth rate, consequently improving biotransformation activity and l-GalOA formation within the first 48 h of the batch process. From our results, we cannot derive which medium component positively affected growth. Since addition of amino acids did not lead to a different growth behavior of *S. cerevisiae*, the strain’s auxotrophies are not the bottleneck*.* However, our results reflect findings from other studies (Roberts et al. 2020). Here, a higher number of cells was reached by increasing the concentration of all components of the synthetic defined medium. Still, biotransformation was stalled within 48 h, even though sufficient sorbitol and d-GalA were present in the medium (Fig. 5). From our results, we cannot pinpoint the reason for this. It was described earlier that low sorbitol levels lead to slower transport kinetics of the Hxt13 transporter (Jordan et al. 2016), possibly limiting supply of NADPH for the reduction of d-GalA. This might be overcome by applying a fed-batch process to maintain sorbitol levels above this threshold, but repeated sorbitol additions were also not successful (data not shown).

To access a wider NADPH-pool for d-GalA reduction, different genetic engineering approaches could be applied. Kim et al. (2018) increased NADPH levels by replacing several NAD^+^-utilizing enzymes from the acetate and ammonium assimilation and pentose phosphate pathways in yeast with equivalent NADPH-generating enzymes. A similar approach was chosen by Verho et al. (2003) to overcome NADPH requirements for pentose fermentation in yeast. They expressed a fungal NADP^+^-dependent glyceraldehyde-3-phosphate dehydrogenase to generate NADPH. Attempts have also been made to address the redox problem by side directed mutagenesis of the respective d-GalA reductase to achieve a higher acceptance of NADH as reducing factor (Harth et al. 2020), widening the pool of utilizable reducing factors for the production of l-GalOA. Furthermore, strategies should be deployed to valorize as many monomeric sugars from SBPP hydrolysate (Jeong et al. 2020) as possible. The presented biotransformation process paves the way for integrated bioprocess development (hydrolysis, biotransformation, and l-GalOA isolation) towards repurposing pectin-rich residues for the production of value-added chemicals. This eventually requires the integration of all genes enabling biotransformation of d-GalA in a robust industrial *S. cerevisiae* strain (Pronk 2002) and also the development of a cost-efficient downstream process. Isolation of the product might be achieved by similar methods as described by Zhou et al. (2018).

## Data Availability

The authors will make available all data (underlying the described findings) without restriction upon request.
